# Editorial: Advanced Deep Learning Methods for Biomedical Information Analysis (ADLMBIA)

**DOI:** 10.3389/fdata.2022.863060

**Published:** 2022-03-01

**Authors:** Yu-Dong Zhang, Shuai Li, Carlo Cattani, Shui-Hua Wang

**Affiliations:** ^1^School of Computing and Mathematical Sciences, University of Leicester, Leicester, United Kingdom; ^2^Department of Electronics and Electrical Engineering, Swansea University, Swansea, United Kingdom; ^3^Engineering School (DEIM), University of Tuscia, Viterbo, Italy

**Keywords:** deep learning, biomedical information analysis, magnetic resonance imaging, social media, omics, surface electromyography, optical character recognition, computed tomography

Due to numerous biomedical information sensing devices, such as Computed Tomography (CT), Magnetic Resonance Imaging (MRI), Ultrasound, Single Photon Emission Computed Tomography (SPECT), and Positron Emission Tomography (PET), to Magnetic Particle Imaging, EE/MEG, Optical Microscopy and Tomography, Photoacoustic Tomography, Electron Tomography, and Atomic Force Microscopy, and so on, a large amount of biomedical information has been gathered in recent years. However, developing advanced imaging methods and computational models for efficient data processing, analysis, and modeling from the collected data remains a challenge.

Deep learning (DL) approaches have been rapidly developed in recent years, both in terms of methodologies and practical applications. DL techniques provide computational models of multiple processing layers to learn and represent data with multiple levels of abstraction. DL allows the capture of intricate structures of large-scale data implicitly and is ideally suited to some of the hardware architectures that are currently available.

The purpose of this Research Topic is to provide a diverse but complementary set of contributions to demonstrate new developments and applications of deep learning and computational machine learning to solve problems in biomedical engineering.

MRI and its related modalities are fundamental imaging tools in biomedical engineering. The method of Germuska et al. is based on the simultaneous acquisition of cerebral blood flow (CBF) and blood oxygen level-dependent (BOLD) weighted images during respiratory modulation of both oxygen and carbon dioxide. The authors present a machine learning implementation for the multi-parametric assessment of dual-calibrated fMRI data. Moreno López et al. evaluate two unsupervised approaches to denoise MRI in the complex image space using the raw information that k-space holds. The first method is based on Stein's Unbiased Risk Estimator, while the second approach is based on a blindspot network, limiting the network's receptive field.

Social media is now another critical tool that can provide information for biomedical analysis. Shah et al. use the advancement of natural language processing algorithms and large-scale data analysis. Their in-depth results show that the proposed method provides a viable solution in less time with the same accuracy compared to traditional methods.

Omics are novel, comprehensive approaches for analyzing the genetic or molecular profiles of humans and other organisms. Hassanzadeh and Wang use an integrated deep belief network to differentiate high-risk cancer patients from the low-risk ones in terms of overall survival. Their study analyzes RNA, miRNA, and methylation molecular data modalities from labeled and unlabeled samples to predict cancer survival and subsequently provide risk stratification.

The surface electromyography (sEMG) assesses muscle function by recording muscle activity above the muscle on the skin. In the paper of Shi et al., the knee flexion angle, hip flexion angle, ankle dorsiflexion angle, and sEMG signals of the seven muscles around the knee of three different data sets (walking data set, running data set, and walking and running mixed data set) are used as input of the 1D CNN. The attention mechanism is added to the network to observe the dimension feature that the network pays more attention to, thereby increasing the interpretability of the model.

Optical character recognition (OCR) technology is a solution for automating data extraction from printed or written text from a scanned document or image file and then converting the text into a machine-readable form to be used for data processing. The paper of Froese et al. builds a script that extracts real-time images from a medication pump and then processes them using Optical Character Recognition to create digital text from the image. This text is then transferred to an ICM + real-time monitoring software parallel with other retrieved physiological data.

A computed tomography (CT) scan combines a series of X-ray images taken from different angles around the body and uses computer processing to create cross-sectional images. The paper of Wang et al. builds a 12-layer convolutional neural network (12l-CNN) as the backbone network. Afterward, PatchShuffle is introduced to integrate with 12l-CNN as a regularization term of the loss function. Their model is named PSCNN. Moreover, multiple-way data augmentation and Grad-CAM are employed to avoid overfitting and locating lung lesions.

There is one review paper included in this Research Topic. Lan et al. introduce the origin, specific working principle, and development history of the generative adversarial network (GAN), various applications of GAN in digital image processing, Cycle-GAN, and its application in medical imaging analysis, as well as the latest applications of GAN in medical informatics and bioinformatics.

The ultimate goal of this Research Topic is to promote research and development of deep learning for multimodal biomedical images by publishing high-quality research articles, reviews, or perspectives, among other article types, in this rapidly growing interdisciplinary field.

## Author Contributions

Y-DZ was the guest editor of this Research Topic and wrote the editorial. SL, CC, and S-HW were guest editors of this Research Topic. All GEs approved the submission of this editorial.

## Conflict of Interest

The authors declare that the research was conducted in the absence of any commercial or financial relationships that could be construed as a potential conflict of interest.

## Publisher's Note

All claims expressed in this article are solely those of the authors and do not necessarily represent those of their affiliated organizations, or those of the publisher, the editors and the reviewers. Any product that may be evaluated in this article, or claim that may be made by its manufacturer, is not guaranteed or endorsed by the publisher.

